# Hierarchical Microporous/Mesoporous Composite Adsorbent for Deep Dehydration of Tetrahydrofuran

**DOI:** 10.3390/ma19122483

**Published:** 2026-06-10

**Authors:** Xiaohui Yu, Jiaying Yu, Naiwang Liu, Xuan Meng, Li Shi

**Affiliations:** International Joint Research Center of Green Energy Chemical Engineering, East China University of Science and Technology, Shanghai 200237, China; y30240139@mail.ecust.edu.cn (X.Y.); yujiaying0517@163.com (J.Y.); mengxuan@ecust.edu.cn (X.M.); yyshi@ecust.edu.cn (L.S.)

**Keywords:** tetrahydrofuran, deep dehydration, hierarchical composite adsorbent, competitive adsorption

## Abstract

The presence of residual moisture in tetrahydrofuran (THF) greatly limits its suitability for moisture-sensitive processes, including polymerization, Grignard chemistry, and fine-chemical production, where the allowable water concentration is generally lower than 10 mg/kg. Here, a hierarchical microporous/mesoporous composite adsorbent was prepared via extrusion molding, combining an LTA-type zeolite microporous framework with an amorphous mesoporous matrix. Characterization by XRD, FTIR, SEM, and pore analysis confirmed that the LTA crystal structure was retained while mesopores provided channels for mass transport. Static dehydration tests showed that the composite reduced THF water content from 70 mg/kg to 8.3 mg/kg, compared to 23.4 mg/kg for commercial 3A molecular sieves. The enhanced performance arises from micropores supplying uniform adsorption sites for deep dehydration and mesopores accelerating diffusion. Water vapor adsorption, kinetic and isotherm analyzes, regeneration, and competitive adsorption experiments indicated improved water accessibility and high selectivity, with kinetics described by a double-exponential model. The adsorbent remained stable over six adsorption–regeneration cycles. These results demonstrate that hierarchical microporous/mesoporous structures effectively achieve deep THF dehydration.

## 1. Introduction

Tetrahydrofuran (THF) plays an important role as a polar, non-proton-donating solvent in polymer-related production, drug-intermediate preparation, and fine-chemical operations, mainly because it can dissolve a broad range of organic substances [1,2,3]. Nevertheless, THF readily takes up small quantities of moisture from the surrounding environment during storage, transfer, or practical use, and this characteristic may impair its suitability for subsequent chemical operations [1]. In polymerization, water can perturb the reaction rate and broaden or shift the molecular-weight distribution [4]. It may also consume or inactivate organomagnesium reagents, thereby lowering the efficiency of Grignard reactions [5]. In addition, changes in solvent polarity caused by water can affect particle dispersion and the crystallization behavior of polymers [6]. For these reasons, THF used in water-sensitive processes normally needs an additional high-efficiency drying treatment, so that the residual moisture concentration can be controlled at less than 10 mg/kg.

Because tetrahydrofuran (THF) contains an ether oxygen within its heterocyclic structure and exhibits moderate polarity, it can interact strongly with water through hydrogen-bond formation [7]. Such interactions give rise to microscopic THF–water association structures. At THF-rich compositions, particularly above 80%, hydrated complexes such as 2THF·H_2_O together with self-associated THF clusters may be generated [8], making the elimination of residual moisture more challenging. The separation of water from THF by ordinary distillation is difficult because of azeotrope formation [9], whereas membrane processes are also problematic since THF may swell or even dissolve a variety of polymeric membrane materials [2]. Compared with these approaches, adsorption is considered a more practical option for deep drying because it can bypass azeotropic restrictions, provide preferential water removal, and operate with lower energy input [10]. However, many commonly used desiccants, including silica gel [11], activated alumina [12], activated carbon [13], metal–organic frameworks [14], and superabsorbent resins [15], still show disadvantages in THF media, such as insufficient uptake, unsatisfactory durability, or high material expense. Zeolitic molecular sieves are more suitable for discriminating between water and THF because their uniform micropores can accommodate small water molecules while sterically blocking the larger solvent molecules [16]. Even so, materials composed only of micropores usually exhibit slow intraparticle transport, especially when liquid THF contains water-related aggregates. This mass-transfer resistance reduces the utilization of internal adsorption centers and consequently weakens the drying efficiency.

A feasible way to address this issue is to introduce multilevel porosity into the adsorbent. In such a structure, microporous regions mainly contribute to selective water uptake, whereas mesoporous channels shorten diffusion distances and improve molecular accessibility [17]. Hierarchical porous materials can be obtained through methods such as topology regulation, templating, post-synthetic etching, or integration with mesoporous components [18]. Previous reports have shown the benefit of this concept in related adsorption systems. For instance, Zhang et al. [19] developed activated alumina/molecular sieve composites for drying compressed air. Dabbawala et al. [20] prepared hierarchical zeolite Y containing connected micro–mesopore networks, and the enhanced water uptake was attributed to more efficient mass transport. Nevertheless, whether similar composite or hierarchical adsorbents can effectively remove trace water from liquid THF has not been fully established.

Although hierarchical zeolites and composite adsorbents have been widely investigated for gas drying and vapor-phase adsorption, their application in deep dehydration of liquid THF remains insufficiently studied. Most previously reported hierarchical zeolites mainly focused on improving pore accessibility through post-synthetic etching or templating strategies, while relatively few studies considered the synergistic combination of microporous selectivity and mesoporous transport enhancement under liquid-phase THF conditions. In addition, many reported hierarchical adsorbents are prepared in powder form, which may suffer from limited mechanical strength, particle agglomeration, and poor applicability in continuous industrial dehydration systems. In this work, an LTA-based hierarchical microporous/mesoporous composite adsorbent was constructed through extrusion molding by integrating a zeolitic microporous phase with an amorphous mesoporous matrix. Compared with conventional powdered hierarchical materials, the extrusion-molded structure not only improves shaping stability and handling performance but also facilitates the formation of interconnected mesoporous transport channels while preserving the intrinsic water selectivity of the LTA microporous framework. Therefore, this study aims to explore the structure–performance relationship of extrusion-formed hierarchical adsorbents for trace-water removal from liquid THF systems.

To address the trade-off between selective adsorption and mass transfer during trace-water removal from THF, a hierarchical microporous/mesoporous composite adsorbent was prepared by combining an LTA-type zeolite microporous phase with an amorphous mesoporous phase through extrusion molding. Its structure was characterized by XRD, FTIR, and pore-structure analysis, and its performance was evaluated by static THF dehydration, water-vapor adsorption, kinetic and isotherm fitting, and regeneration tests. The aim of this study is to clarify how the hierarchical structure influences deep dehydration of THF and to provide an experimental basis for the design of efficient adsorbents for trace-water removal from organic solvents.

## 2. Experimental Section

### 2.1. Reagents and Instruments

All chemicals used in this work were of analytical purity unless otherwise specified. Sodium silicate, sodium hydroxide, aluminum hydroxide, potassium chloride, concentrated nitric acid, silica sol, polyethylene glycol, ethanol, and tetrahydrofuran (THF) were used as received. Commercial 3A, 4A, and 5A zeolites were supplied by China National Pharmaceutical Group Chemical Reagent Co., Ltd. (Shanghai, China). The atmosphere-controlled glove box used in the experiments was a VGB-4-C unit from Changshu Tongrun Electronic Technology Co., Ltd. (Changshu, China)., and the water content was determined using an MKC-710B Karl Fischer moisture analyzer manufactured by Kyoto Electronics Manufacturing Co., Ltd. (Kyoto, Japan).

### 2.2. Preparation of Adsorbent

Preparation of the Microporous Component: The microporous component was synthesized via a hydrothermal method in this study. First, sodium silicate was formulated into an aqueous solution with a relative density of 1.2, which was allowed to stand, and the supernatant was collected as the silicon source. Aluminum hydroxide and sodium hydroxide were blended in a mass ratio of 1.2:1 and then heated to dissolve, so as to prepare a sodium aluminate solution that served as the aluminum source. The silicon source, aluminum source, and sodium hydroxide mentioned above were accurately weighed in line with a molar ratio of Al_2_O_3_:SiO_2_:Na_2_O:H_2_O = 1:2:3:185, and then stirred continuously at 80 °C for 30 min to form a homogeneous gel. The gel was transferred into a high-pressure autoclave, first undergoing crystallization at 90 °C for 1 h, and then heated to 102 °C for an additional 4 h of further crystallization. After natural cooling to room temperature, vacuum filtration was carried out, and the filter cake was rinsed with deionized water until the pH value of the filtrate was approximately 9. The obtained filter cake was dried at 120 °C for 12 h. After drying, the filter cake was added to a 0.2 mol/L potassium chloride solution at a solid–liquid ratio of 1 g:10 mL, and stirred at 60 °C for ion exchange. After exchange, filtration and washing were conducted to remove free potassium ions, the sample was then dried at 120 °C for 12 h and activated by calcination at 550 °C for 4 h, yielding the microporous component.

Preparation of the Mesoporous Component: The mesoporous phase was prepared using a sol–gel route. To form the aluminum precursor sol, 3.0 g of aluminum hydroxide was added to 45 mL of deionized water, after which 10 mL of concentrated nitric acid with a concentration of approximately 65 wt% was introduced dropwise. The suspension was kept at 80 °C for 2 h under continuous agitation at 500 rpm until a clear aluminum-containing sol was obtained. In a separate vessel, 20.0 g of silica sol containing 40 wt% SiO_2_ was mixed with 60 mL of deionized water. Polyethylene glycol (PEG, 0.8 g) was then incorporated into this diluted silica precursor at 60 °C, serving as a soft porogenic agent to promote mesopore formation during gelation. The silica-based precursor was gradually fed into the aluminum sol at about 1 mL/min while stirring was maintained. After complete addition, the mixed sol was subjected to thermal aging at 75 °C for 18 h, during which a uniform milky gel was generated. The obtained gel was rinsed three times with an ethanol/water solution at a volume ratio of 1:1 to eliminate remaining soluble species. Finally, the washed solid was oven-dried at 80 °C for 12 h, giving the mesoporous component.

Molding of the Microporous/Mesoporous Composite Adsorbent: To obtain the composite adsorbent, predetermined amounts of the microporous component and mesoporous component were first blended with 0.2 g of sesbania powder. Afterward, 5 mL of diluted nitric acid solution with a concentration of 5 wt% was introduced as a shaping liquid, and the wet mixture was kneaded thoroughly until a plastic paste suitable for extrusion was formed. The paste was then shaped using a screw-type extruder. The extrudates were dried at 120 °C overnight and then thermally treated in a muffle furnace at 550 °C for 4 h. During calcination, the temperature was increased stepwise: first to 150 °C within 15 min, then to 300 °C within 30 min, and finally to 550 °C over 125 min. After natural cooling, the calcined solid was ground and screened to collect particles in the 20–40 mesh range, which were used as the final micro–mesoporous composite adsorbent. A series of samples with different proportions of the two porous phases was obtained by adjusting the initial feed composition while keeping the shaping and heat-treatment conditions unchanged. In this paper, these samples are denoted as Micro/Meso-x-y, where “x” and “y” represent the theoretical mass fractions of the prepared microporous component and mesoporous component, respectively.

### 2.3. Static Adsorption Experiment

For each static dehydration test, 1.0 g of the pretreated adsorbent was contacted with 30 mL of THF containing 70 mg/kg water in a sealed reagent bottle. The adsorbent particles were previously screened to 20–40 mesh and activated in a muffle furnace at 300 °C for 4 h. The sealed bottle was transferred to a glove box under a nitrogen atmosphere and kept at 25.0 ± 0.5 °C for 18 h to allow the adsorption process to approach equilibrium. After treatment, the residual moisture concentration of THF was measured by coulometric Karl Fischer titration.

### 2.4. Characterization of Adsorbent

Powder X-ray diffraction analysis was performed to examine the crystalline characteristics and phase constitution of the adsorbent samples. The measurements were carried out using a Tonda 5000 diffractometer equipped with a Cu Kα source with a wavelength of 0.15406 nm. Diffraction signals were acquired within the 2θ interval of 5–80°, and the scan step/rate was set to 0.02° s^−1^.

The textural parameters of the composite adsorbent, including surface area and pore-size characteristics, were measured with a JWGB-JQ200C surface-area/pore-size analyzer manufactured by Beijing Jingwei Gaobo Technology Co., Ltd. (Beijing, China). The surface area values were derived from nitrogen adsorption data using both the BET and Langmuir models [21]. The overall pore volume was estimated from the adsorbed amount at a relative pressure of P/P_0_ = 0.99. Micropore volume was evaluated by the t-plot method, whereas the mesopore contribution was obtained by subtracting the micropore volume from the overall pore volume.

To further characterize the microporous structure of the adsorbents, CO_2_ adsorption–desorption measurements were performed as a complementary analysis [22]. CO_2_ isotherms were collected at 273 K using an ASAP 2460 surface area and pore size analyzer (Micromeritics, Norcross, GA, USA). The micropore parameters were then evaluated using the Horvath–Kawazoe (HK) method and density functional theory (DFT) model [23]. Specifically, the micropore volume and median pore size were calculated by the HK method, while the pore area and pore-volume distribution were derived from the CO_2_-DFT model. CO_2_ adsorption–desorption analysis provides further insight into the microporous structure of the adsorbents and helps clarify the relationship between microporosity and adsorption performance.

The framework features and water-affinity functional moieties of the adsorbent were investigated by FTIR analysis. Spectra were obtained on a Thermo Scientific Nicolet iN10 infrared spectrometer (Thermo Fisher Scientific Inc, Waltham, MA, USA) at a resolution of 4 cm^−1^. Data acquisition was performed within the wavenumber region of 4000–500 cm^−1^.

In this work, the surface micromorphology of the adsorbent was characterized using a field-emission scanning electron microscope (Carl Zeiss GeminiSEM 500, Oberkochen, Germany). Before testing, the adsorbent powder was uniformly affixed to the conductive adhesive on the sample stage, and loosely bound excess particles were gently removed by purging with a rubber suction bulb. The sample surface was then sputter-coated with a platinum-alloy to improve electrical conductivity and suppress charging artifacts. All images were acquired under the secondary electron (SE) imaging mode to record the surface morphological characteristics of the adsorbent.

### 2.5. Water Vapor Adsorption Curve and Model Fitting

Water-vapor adsorption measurements were performed to evaluate the adsorption characteristics of the adsorbent, using instruments operated according to gravimetric and volumetric principles. The time-dependent water-uptake profiles were further interpreted with kinetic models, including the pseudo-first-order, pseudo-second-order, and double-exponential equations [24,25]. To reveal the adsorption mechanism in the microporous/mesoporous composite adsorbent, the equilibrium uptake data were correlated using a hybrid isotherm model composed of the Dubinin–Astakhov and Toth equations [26,27]. Through these analyzes, both the adsorption-rate behavior and equilibrium capacity of water in the hierarchical porous structure could be assessed.

#### 2.5.1. Steam Adsorption Experiment

(a) Volumetric Principle

Water-vapor uptake measurements were carried out on a static volumetric adsorption instrument supplied by Beijing JWGB Sci. & Tech. Co., Ltd. (Beijing, China). Before testing, approximately 0.1–0.2 g of adsorbent was placed in the analysis tube and activated under vacuum at 300 °C for 3 h to remove pre-adsorbed species. After pretreatment, the isothermal adsorption data were collected at 25 °C by gradually changing the relative pressure from 0.1 to 1.0.

(b) Gravimetric Principle

Adsorption uptake in the gravimetric test was evaluated from the variation in adsorbent mass during the adsorption process. This technique also enables the acquisition of isobaric uptake-rate profiles, which can be further used to analyze adsorption kinetics. In the present work, the measurements were performed with a multi-station gravimetric gas–vapor adsorption apparatus supplied by Beijing Bes Tech Instrument Technology Co., Ltd. (Beijing, China).

#### 2.5.2. Dynamics Fitting

Adsorption kinetic models are frequently utilized to analyze the mass transfer mechanism in adsorbent materials and the key parameters governing the adsorption kinetics [24]. In this study, three kinetic models based on different adsorption mechanisms (pseudo-first-order model, pseudo-second-order model, and double-exponential model) were employed to analyze the kinetic behavior of the water adsorption.

The pseudo-first-order (PFO) kinetic model presumes that the adsorption rate bears a proportional relationship with the number of unoccupied adsorption active sites on the adsorbent, which is generally applicable to physical adsorption phenomena [28]. Its equation is as follows:

(1) $$\mathrm{lg}\left(q_{e}-q_{t}\right){=\mathrm{lgq}}_{e}-\frac{k_{1}t}{2.303}$$

where $q_{e}$ (mg/g) is the equilibrium adsorption capacity, $q_{t}$ (mg/g) is the adsorption capacity at time t (min), $k_{1}$ (min^−1^) is the pseudo-first-order rate constant.

The pseudo-second-order (PSO) kinetic model is founded on the assumption that the adsorption rate is proportional to the square of the amount of unoccupied active sites in the adsorbent. This model is commonly employed to characterize chemical adsorption, which proceeds via the sharing of valence bonds or electron exchange between the adsorbent and the adsorbate [29]. Its equation is as follows:

(2) $$\frac{t}{q_{t}}=\frac{1}{k_{2}q_{e}^{2}}+\frac{t}{q_{e}}$$

where $q_{e}$ and $q_{t}$ have the same meanings as those in the pseudo-first-order model, $k_{2}$ (g mg^−1^ min^−1^) is the pseudo-second-order rate constant, and t (min) is the adsorption time.

The double-exponential model consists of two exponential functions that describe the relationships between adsorption, coverage, and the rate of adsorption layer formation. Its equation is as follows:

(3) $$q_{t}{\;=q}_{e}-A_{1}e^{{-k}_{3}t}-A_{2}e^{{-k}_{4}t}$$

where $q_{t}$ (mg/g) is the adsorption capacity at time t (min), $k_{3}$ and $k_{4}$ (s^−1^) are the adsorption rate constants of the two stages, respectively, and $A_{1}$ and $A_{2}$ is the pre-exponential factor.

#### 2.5.3. Water Vapor Adsorption Isotherm Model

The shape of a water-vapor adsorption isotherm can reveal how water molecules interact with the surface of an adsorbent. By applying suitable isotherm equations, it is possible to distinguish different adsorption behaviors, such as single-layer coverage or multilayer accumulation, and to obtain mechanistic information about the underlying water-uptake process. In this work, the adsorption isotherms of microporous materials, mesoporous materials, and microporous/mesoporous composite adsorbents were individually fitted using the Dubinin–Astakhov (D–A) model, the Toth model, and their combined model, respectively.

The Dubinin–Astakhov (D–A) model is a classical physical adsorption model developed on the basis of the micropore adsorption potential theory. It has been widely adopted to describe the adsorption behavior of gases or vapors on microporous materials, especially for adsorption systems governed by the micropore volume-filling mechanism. Accordingly, it stands as the preferred model for fitting adsorption isotherms of microporous solid adsorbents such as molecular sieves, microporous zeolites, and microporous carbon materials [26]. The model equation is given as follows:

(4) $${V=V}_{0}\exp\left[-{\left(\frac{\mathrm{RTln}\frac{P_{0}}{P}}{E}\right)}^{n}\right]\;$$

where V (cm^3^/g STP) is the equilibrium adsorption capacity, $V_{0}$ (cm^3^/g STP) is the limiting micropore adsorption capacity, E (J/mol) is the characteristic adsorption energy reflecting the interaction strength between adsorbate and adsorbent, and n is the structural heterogeneity parameter used to characterize pore regularity and the uniformity of the adsorption potential distribution.

The Toth model is a semi-empirical adsorption model applicable to adsorption systems featuring heterogeneous surfaces, originally proposed by Toth in 1971. By incorporating a parameter that characterizes surface heterogeneity, it overcomes the limitation of the Langmuir model, which is only valid for ideal homogeneous surfaces [27]. This model provides a satisfactory description of the adsorption behavior of gases and vapors on irregular surfaces, such as those of mesoporous materials and supported materials, making it a widely employed model for fitting water vapor adsorption isotherms of mesoporous materials. The model equation is given below:

(5) $$V=\frac{V_{m}\mathrm{bx}}{{\left[1+{\left(\mathrm{bx}\right)}^{t}\right]}^{1/t}}$$

where V (cm^3^/g STP) denotes the equilibrium adsorption capacity; $V_{m}$ (cm^3^/g STP) represents the saturated adsorption capacity; b is the dimensionless adsorption affinity constant, with a larger value corresponding to stronger adsorption affinity; and t is a parameter characterizing the uniformity of the adsorption site energy distribution.

The adsorption isotherms of the microporous/mesoporous composite adsorbents were fitted using a combined D–A + Toth model, expressed as follows:

(6) $${V=V}_{0}\exp\left[-{\left(\frac{\mathrm{RTln}\frac{P_{0}}{P}}{E}\right)}^{n}\right]+\frac{V_{m}\mathrm{bx}}{{\left[1+{\left(\mathrm{bx}\right)}^{t}\right]}^{1/t}}\;$$

#### 2.5.4. Diffusion Coefficient

The diffusion coefficient was obtained by fitting and mathematically processing the adsorption kinetic profile. Because the adsorbents tested in this work were all in powder form, a spherically symmetric diffusion model was adopted for the calculation. The corresponding expression is given below [30]:

(7) $$\frac{M_{t}}{M_{\infty }}=1-\frac{6}{\pi^{2}}\overset{\infty }{\underset{n=1}{\sum }}\frac{1}{n^{2}}\exp\left(-\frac{{\mathrm{Dn}}^{2}\pi^{2}t}{r^{2}}\right)$$

At the early adsorption stage (Mt/M_∞_ < 0.2), the expression can be approximated as follows [31]:

(8) $$\frac{M_{t}}{M_{\infty }}=\frac{r}{6}\sqrt{\frac{\mathrm{Dt}}{\pi }}$$

The value can be obtained from the slope, k, of the early linear region:

(9) $$D=\frac{\pi }{36}{\left(\mathrm{kr}\right)}^{2}$$

where $M_{t}$ represents the adsorption amount at time t, $M_{\infty }$ represents the final adsorption amount when equilibrium is reached, D is the diffusion coefficient, and r is the average particle size of the powder.

### 2.6. Competitive Adsorption Experiment

To elucidate the competitive adsorption behavior of water and tetrahydrofuran (THF) on the hierarchical microporous/mesoporous adsorbents, dynamic breakthrough experiments were conducted at 25 °C using a multicomponent competitive adsorption breakthrough analyzer manufactured by Beijing Best Instruments Technology Co., Ltd. (Beijing, China). The feed stream consisted of 0.30 vol% THF and 1.56 vol% water vapor, with N_2_ as the balance gas.

The activated adsorbent powder was packed into a breakthrough column, and the mixed gas was passed through the adsorbent bed. The effluent was subsequently introduced into an online mass spectrometer to continuously monitor the temporal evolution of each component concentration at the column outlet. The dynamic adsorption process was represented by breakthrough curves, in which the outlet-to-inlet concentration ratio, C_out_/C_in_, was plotted as a function of time normalized by adsorbent mass (s g^−1^). In this work, the breakthrough point was defined as the time at which the outlet adsorbate concentration reached 5% of the inlet concentration. The half-saturation point was defined as the time corresponding to C_out_/C_in_ = 0.50, whereas the saturation point was defined as the time at which C_out_/C_in_ reached 0.95. These characteristic points provide quantitative criteria for assessing the dynamic adsorption capacity of the adsorbents toward different components and for evaluating their competitive adsorption behavior.

## 3. Results and Discussion

### 3.1. XRD Analysis

**Figure 1:**
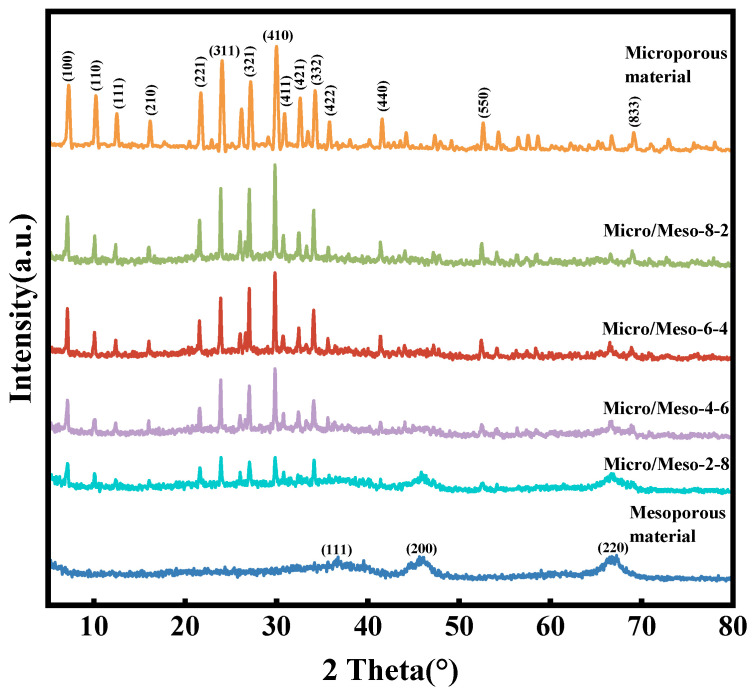
X-ray patterns of the prepared adsorbents.

X-ray diffraction was performed to analyze the phase structure of the prepared adsorbents. As shown in Figure 1, the microporous sample exhibits typical LTA-related diffraction peaks at 2θ = 12.4°, 16.1°, 21.6°, 23.9°, 27.1°, and 29.9°, corresponding to the (111), (210), (221), (311), (321), and (410) planes, respectively. No additional crystalline phases are detected, indicating that the microporous material has high phase purity and retains the LTA framework. In contrast, the mesoporous material shows only weak, broad diffraction features, consistent with its amorphous nature. For the composite adsorbents, the LTA peak intensity decreases as the mesoporous fraction increases, mainly due to dilution of the crystalline microporous phase [32]. No new peaks or obvious peak shifts appear in the composite samples, suggesting that the two porous components are physically integrated and that the original framework structures remain intact.

### 3.2. Pore Structure Analysis

**Figure 2:**
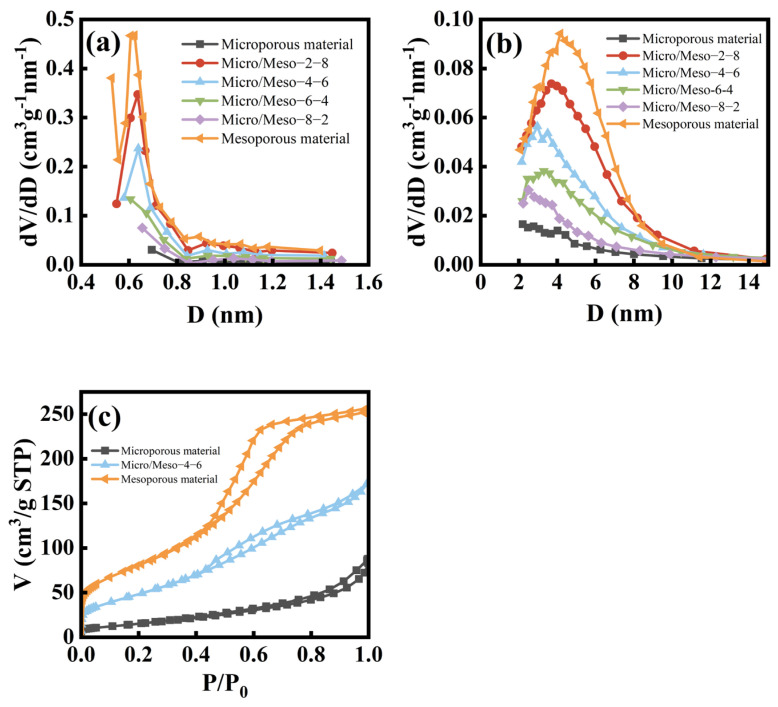
(**a**) Micropore pore size distribution plot, (**b**) Mesopore pore size distribution plot, and (**c**) N_2_ adsorption–desorption isotherm.

Nitrogen adsorption–desorption isotherms and pore-size distributions provide essential evidence for characterizing the pore structure of adsorbents, particularly for identifying the presence and distribution characteristics of mesopores. As shown in Figure 2c, the mesoporous material exhibits a typical type IV isotherm with a pronounced hysteresis loop, and capillary condensation occurs in the P/P_0_ range of 0.4–0.8, confirming the presence of mesoporous channels [33]. The composite samples combine features of both microporous and mesoporous materials. Although XRD confirms that the microporous phase is an LTA-type zeolite, the N_2_-based surface area values are relatively low, and the microporous signature is weak. This is expected because the kinetic diameter of N_2_ (~0.36 nm) is slightly larger than the pore opening of LTA zeolite (~0.30 nm) [34], making it difficult for N_2_ to access the micropores at 77 K. To better characterize the microporous structure, CO_2_ adsorption–desorption measurements were further conducted to determine the specific surface area, pore volume, and pore-size distribution [35]. Owing to its better compatibility with the LTA pore aperture, CO_2_ can more effectively access the micropores. As shown in Table 1, the microporous materials exhibit high micropore surface areas and large micropore volumes, with pore sizes mainly within the typical LTA micropore range. These results confirm the presence of microporous components and indicate that N_2_ adsorption alone cannot fully capture the microporous features because of probe-molecule diffusion limitations and pore-accessibility constraints.

The pore-size distributions in Figure 2a,b show that the composite samples contain pores spanning approximately 0.4–10 nm, indicating the coexistence of micro- and mesopores. Table 2 shows that increasing the mesoporous fraction leads to higher N_2_-derived specific surface areas and total pore volumes for the composite adsorbents. This increase is attributed to the abundant pore channels, large surface area, and high porosity of the mesoporous component, which provide more accessible pore space and make the overall textural properties of the composites closer to those of mesoporous materials. Thus, the composite adsorbents are better described as hierarchical microporous/mesoporous materials: their mesoporous features are characterized by N_2_ adsorption, while their microporous features are confirmed by CO_2_ adsorption. For deep dehydration of organic solvents, the N_2_-derived textural properties are more relevant for evaluating mass-transfer behavior and guiding subsequent dehydration experiments.

### 3.3. FTIR Analysis

**Figure 3:**
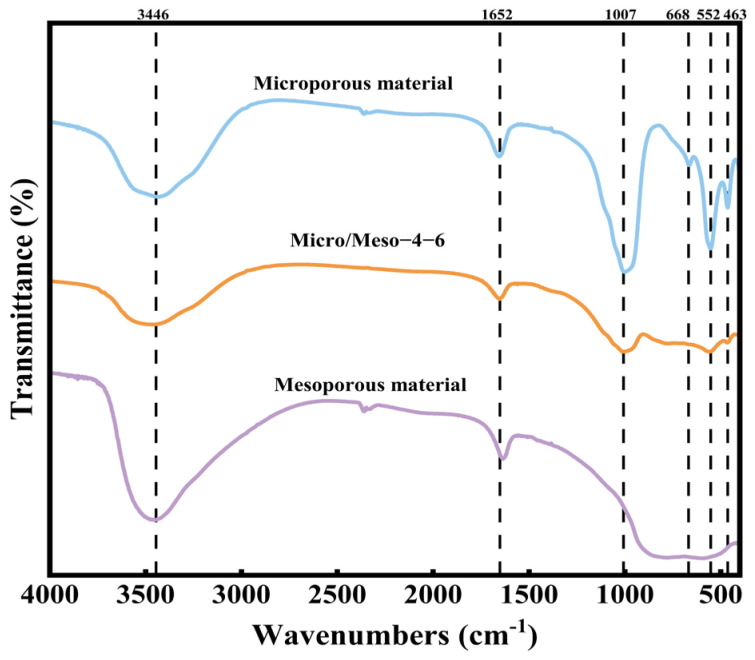
FTIR spectra of different adsorbents.

FTIR spectra were recorded to examine the functional groups and framework vibrations of the prepared adsorbents. As shown in Figure 3, the bands at 3446 and 1652 cm^−1^ are related to surface –OH vibrations [36]. The peaks at 463, 668, and 1007 cm^−1^ correspond to Si–O–Al framework vibrations and asymmetric stretching of internal tetrahedral units [37,38]. The band near 552 cm^−1^ is assigned to the double four-ring (D4R) structure of LTA zeolite [38]. The microporous material exhibits typical LTA framework features, whereas the mesoporous material mainly shows Si–O/Al–O and hydroxyl-related vibrations. In the composites, the main bands of both phases remain visible, with no obvious shifts or new absorption bands, suggesting physical combination and largely unchanged chemical environments.

### 3.4. SEM Analysis

**Figure 4:**
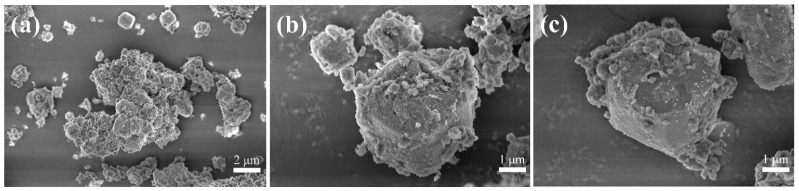
SEM images of Micro/Meso-4-6 (**a**) Low-magnification image showing the overall morphology of the composite adsorbent; (**b**) High-magnification image showing the surface of zeolite crystals coated with mesoporous components; (**c**) High-magnification image showing the aggregated mesoporous phase on the zeolite surface and the encapsulated composite structure.

Figure 4 depicts the SEM morphologies of the Micro/Meso-4-6 composite adsorbent. At low magnification, the primary particles of the composite adsorbent exhibit a well-defined cubic octahedral morphology, characteristic of potassium-type A zeolite (LTA topology). The crystals possess a relatively uniform size with slight interparticle agglomeration, and the overall structure is loose and porous, verifying that the microporous zeolite component, serving as the core framework, retains excellent crystallinity and regular morphological features. Examination at high magnification further reveals that the zeolite crystal surfaces are not smooth and dense; instead, they are uniformly loaded and coated with abundant nanoscale mesoporous components. These mesoporous phases are densely distributed on the zeolite surfaces and within intercrystalline gaps, without disrupting the inherent octahedral crystal profile. As such, the mesoporous material heterogeneously encapsulates the microporous framework and forms irregular aggregates on its surface [33], rendering the overall surface rough. This structural feature confirms the formation of an encapsulated composite architecture, in which the mesoporous phase tightly surrounds the microporous zeolite crystals. Such a hierarchical structure not only preserves the superior highly selective adsorption of water molecules by potassium-type A zeolite but also optimizes pore diffusion via the mesoporous layer, thereby facilitating enhanced mass transfer rate and cyclic operational stability of the adsorbent.

### 3.5. Comparison of Water Adsorption Capacities

**Figure 5:**
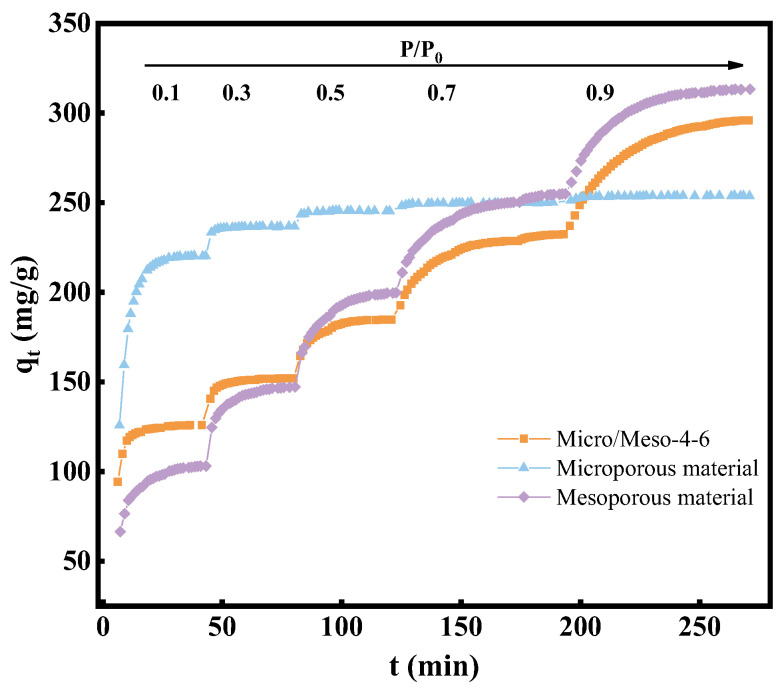
Water adsorption equilibrium curves of different adsorbents at different partial pressures.

Water-vapor adsorption isotherms measured by the gravimetric method are shown in Figure 5. The overall equilibrium uptake follows the order mesoporous material > Micro/Meso-4-6 > microporous material. In the low-pressure region (P/P_0_ < 0.5), uptake is mainly governed by micropore filling. Under these conditions, the microporous materials show strong water adsorption because of their smaller pore size and stronger adsorption potential. The mesoporous material exhibits the lowest uptake in this region, as adsorption is primarily governed by weak intermolecular interactions, including van der Waals forces and weak hydrogen bonding between THF–water molecules and the pore surface functional groups. These interactions are significantly weaker than the capillary condensation mechanisms dominant in microporous structures. The composite samples show somewhat lower uptake than the pure microporous materials at low pressure because part of the adsorbent mass is replaced by the mesoporous phase. In the medium- to high-pressure region (0.5 < P/P_0_ < 0.9), the uptake of the microporous materials approaches a plateau, whereas the mesoporous material shows a sharp increase due to capillary condensation. In the composites, capillary condensation in the mesoporous domain gradually compensates for the saturation of the microporous domain, so the total uptake becomes comparable to or even higher than that of the pure microporous materials. At P/P_0_ = 0.9, the mesoporous material exhibits the highest uptake because capillary condensation is most pronounced. Overall, the hierarchical structure combines efficient low-pressure capture by micropores with high-pressure storage and transport contributions from mesopores, giving the composites a more balanced adsorption profile across the full humidity range.

### 3.6. Static Dehydration Performance

**Figure 6:**
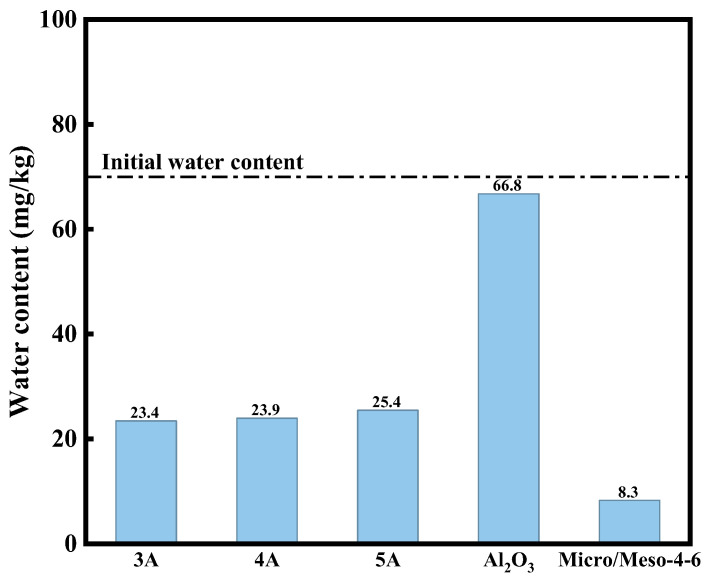
Dehydration performance of commercial adsorbents for tetrahydrofuran (Initial water content: 70 mg/kg).

Figure 6 presents a preliminary comparison of the static dehydration performance of representative commercial adsorbents, including 3A, 4A, 5A zeolites and alumina, under identical experimental conditions. Among these materials, 3A, 4A, and 5A reduce the water content of THF to approximately 20–30 mg/kg. However, none of them lowers the water content below 10 mg/kg under the present conditions. The limited deep-drying performance is likely related to the diffusion resistance associated with purely microporous structures. Compared with conventional microporous adsorbents, the Micro/Meso-4-6 material exhibited improved deep-dehydration performance, suggesting that the introduction of mesoporous channels may facilitate mass transport and reduce diffusion resistance during adsorption.

**Figure 7:**
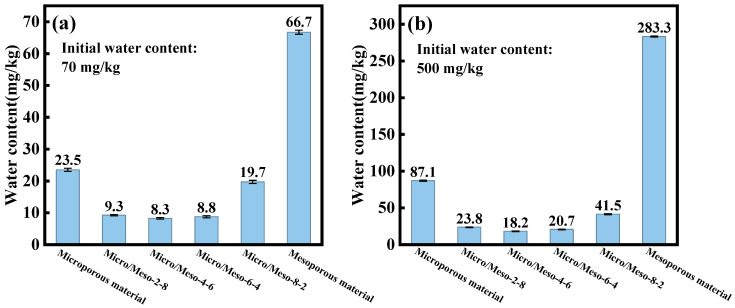
Dehydration performance of different adsorbents for tetrahydrofuran: (**a**) initial water content of 70 mg/kg; (**b**) initial water content of 500 mg/kg.

Figure 7 shows that the pure microporous materials outperform the pure mesoporous material in terms of final dehydration depth, while the composite samples show further improvement, indicating that a hierarchical microporous/mesoporous structure is beneficial for deep drying of THF. As the mesoporous fraction increases, performance first improves and then declines. This trend suggests that introducing an appropriate amount of mesoporosity can relieve diffusion limitations and increase the accessibility of water molecules to the adsorption sites, whereas excessive mesoporous content dilutes the microporous phase and weakens selective water capture. At an initial water content of 70 mg/kg, Micro/Meso-2-8, Micro/Meso-4-6, and Micro/Meso-6-4 all reduce the water content of THF to below 10 mg/kg, with Micro/Meso-4-6 giving the lowest final value of 8.3 mg/kg. When the initial water content is increased to 500 mg/kg, Micro/Meso-4-6 again gives the best result, lowering the water content to 18.1 mg/kg. On this basis, a microporous/mesoporous mass ratio of 4:6 was selected as optimal in this work.

The superior dehydration performance of the composite adsorbents can be attributed to the synergistic roles of the microporous and mesoporous domains. The microporous phase provides selective adsorption sites for water, whereas the mesoporous phase supplies additional mass-transfer pathways, shortens diffusion distances, and improves the accessibility of water molecules to the microporous sites. The hierarchical structure therefore helps balance dehydration depth and transport efficiency in liquid-phase THF.

The ranking observed in liquid-phase THF dehydration is Micro/Meso-4-6 > microporous material > mesoporous material. This order differs from the ranking based solely on gas-phase water-vapor uptake, showing that competitive adsorption and mass-transfer effects in liquid THF strongly influence practical dehydration performance. In the mesoporous material, the larger pore size and surface hydroxyl groups may make THF more accessible to the adsorption surface, which weakens preferential water uptake. Pure microporous materials provide better molecular sieving toward water, but they suffer from restricted internal diffusion. The composite materials partially reconcile these two requirements by combining selective micropores with mesoporous transport pathways, which explains their superior performance in liquid-phase deep dehydration.

### 3.7. Competitive Adsorption

**Figure 8:**
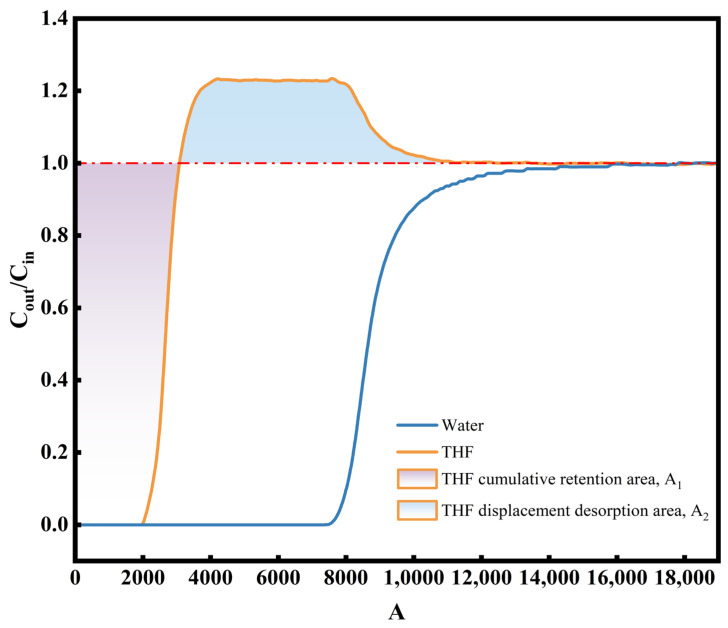
Water and tetrahydrofuran adsorption breakthrough curves on the microporous/mesoporous composite adsorbent (The red dashed line indicates C_out_/C_in_ = 1, representing the inlet concentration and the breakthrough point of the adsorbate).

To evaluate the deep-dehydration capability of Micro/Meso-4-6, which exhibited the best dehydration performance, dynamic breakthrough experiments were performed using a multicomponent THF/H_2_O/N_2_ gas mixture. This experiment provides a representative simulation of the dynamic separation behavior of the adsorbent under conditions in which the organic component and water coexist during THF dehydration. The adsorption–breakthrough curves are shown in Figure 8, and the corresponding experimental results are summarized in Table 3. The results demonstrate that the adsorbent exhibits pronounced preferential water adsorption in the competitive THF/H_2_O binary system. When the breakthrough time was normalized by adsorbent mass, the 5% breakthrough point, 50% half-saturation point, and 95% saturation point for THF were 2159.7, 2664.7, and 3019.5 s·g^−1^, respectively. By contrast, the corresponding 5% breakthrough point, 50% half-saturation point, and 95% saturation point for water were 7850.3, 8655.4, and 11,384.7 s·g^−1^, respectively. The characteristic breakthrough times of water were markedly delayed relative to those of THF, indicating that the material surface interacts more strongly with water molecules. As a result, Micro/Meso-4-6 can preferentially capture trace water from the mixed stream, whereas THF penetrates the adsorption bed at an earlier stage.

In terms of adsorption capacity, this adsorbent also exhibited strong hydrophilicity. The measured adsorption uptake of THF was only 7.433 mL·g^−1^ STP, whereas that of water reached 234.008 mL·g^−1^ STP, far exceeding the THF uptake. In addition, the THF/H_2_O separation factor was only 0.1652, i.e., less than unity, indicating that under competitive adsorption conditions, the adsorbent does not preferentially adsorb THF but instead shows substantially higher adsorption selectivity toward water. This result is consistent with the design objective of a deep-dehydration adsorbent: to achieve efficient water removal from THF-containing systems by enhancing the selective capture of trace water.

The competitive adsorption mechanism can be further elucidated by analysing the breakthrough curves. As shown in Figure 8, after the breakthrough, the normalized outlet concentration of THF (C_out_/C_in_) transiently exceeded unity before gradually decreasing and approaching 1. In parallel, the normalized outlet concentration of water increased sharply at a substantially higher time region, expressed in s·g^−1^. To quantify this process, C_out_/C_in_ = 1 was taken as the baseline, and the shaded regions on both sides of the THF breakthrough curve were integrated. The area between the curve and the baseline in the region where C_out_/C_in_ < 1 can be expressed as:

(10) $$A_{1}=\int_{t_{0}}^{t_{1}}\left(1-\frac{C_{out}}{C_{in}}\right)dt$$

The integrated area of this region, denoted as A_1_, was 2628.6 s·g^−1^. This value corresponds to the cumulative amount of THF retained by the adsorption bed during the initial stage of adsorption and reflects the process by which THF molecules enter the pore channels and occupy a fraction of the adsorption sites. By contrast, the area between the curve and the baseline in the region where C_out_/C_in_ > 1 can be expressed as:

(11) $$A_{2}=\int_{t_{1}}^{t_{2}}\left(\frac{C_{out}}{C_{in}}-1\right)dt$$

The integrated area of this region, denoted as A_2_, was 1254.5 s·g^−1^. This value represents the excess effluent amount of THF, corresponding to the “overshoot” peak generated as THF molecules initially adsorbed on the material surface were progressively displaced by water molecules during the subsequent competitive adsorption process. Because A_1_ is markedly larger than A_2_, only part of the initially retained THF contributed to the subsequent overshoot behavior. The remaining fraction likely eluted gradually during the later breakthrough stage or remained weakly retained within the pore structure. It should be noted that A_1_ and A_2_ represent normalized breakthrough-curve integration parameters with units of s·g^−1^. Conversion into absolute adsorption amounts requires the incorporation of inlet concentration, gas flow rate, and molar-volume relationships. In the present study. Instead, a portion of the retained THF likely eluted gradually during the normal breakthrough process, while a small fraction may have remained within the pore channels. These results indicate that, although the material exhibits a certain degree of initial affinity toward THF, water competes much more strongly for the active adsorption sites. Consequently, adsorbed THF can be effectively displaced by water, demonstrating a typical competitive displacement mechanism in which the strongly adsorbed component, water, replaces the weakly adsorbed component, THF.

Furthermore, the competitive breakthrough behavior provides additional insight into the interaction between THF molecules and the hierarchical pore structure of the adsorbent. Although the material exhibits strong preferential adsorption toward water, the breakthrough curves indicate that THF molecules are initially retained within the adsorption bed before being partially displaced by water. Quantitative integration of the breakthrough curve showed that the retained THF amount corresponding to the adsorption region (A1 = 2628.6 s·g^−1^) was substantially larger than the overshoot displacement region (A2 = 1254.5 s·g^−1^). This result confirms that a fraction of THF remained adsorbed within the pore network even after competitive displacement by water.

Considering the relatively low equilibrium THF uptake (7.433 mL·g^−1^ STP) compared with water (234.008 mL·g^−1^ STP), the interaction between THF and the adsorbent can be regarded as comparatively weak and reversible. Because THF possesses a larger kinetic diameter and lower polarity than water, it is unlikely to strongly occupy the narrow high-energy micropores that dominate water adsorption. Instead, THF molecules are more likely to be temporarily accommodated within mesoporous channels or located near pore entrances through weak van der Waals interactions. By contrast, water molecules can effectively access the microporous adsorption sites owing to their smaller molecular size and stronger polarity, thereby competitively replacing the weakly adsorbed THF molecules during the subsequent adsorption process.

This interpretation is also consistent with the hierarchical pore design of the Micro/Meso-4-6 adsorbent. In this structure, micropores primarily provide strong adsorption potential fields and selective adsorption sites for water capture, whereas mesopores mainly function as transport channels that facilitate molecular diffusion and mass transfer. The transient THF adsorption observed during breakthrough therefore likely originates from short-term occupation of mesoporous regions and external surface sites rather than strong adsorption within the microporous domains responsible for deep dehydration.

Moreover, because the retained THF amount was limited and a large fraction of THF could be displaced during competitive adsorption, THF adsorption is not expected to cause severe pore blocking or irreversible occupation of active adsorption sites. This behavior is beneficial for maintaining the long-term stability and regenerability of the adsorbent under cyclic dehydration conditions. The results therefore suggest that the hierarchical microporous/mesoporous architecture not only enhances water-selective adsorption, but also mitigates the adverse effects of organic molecule accumulation during dynamic THF dehydration.

For the microporous/mesoporous composite adsorbent developed in this study, the above competitive adsorption results demonstrate that the material not only possesses a high water adsorption capacity in the THF/H_2_O binary system, but also exhibits excellent dynamic dehydration performance and stable mass-transfer characteristics. In general, micropores are considered to provide strong confinement effects and abundant high-energy adsorption sites, which constitute a key structural basis for preferential water adsorption and deep dehydration. Mesopores, by contrast, can effectively reduce mass-transfer resistance, shorten diffusion pathways, and accelerate the migration of molecules toward active adsorption sites in multicomponent systems. Therefore, considering both the hierarchical pore architecture of this sample and its excellent performance in the dynamic breakthrough experiments, it can be inferred that the integrated construction of micropores and mesopores is beneficial for enhancing the overall dehydration performance of the adsorbent in THF-containing systems.

### 3.8. Adsorption Kinetics and Model Fitting

**Figure 9:**
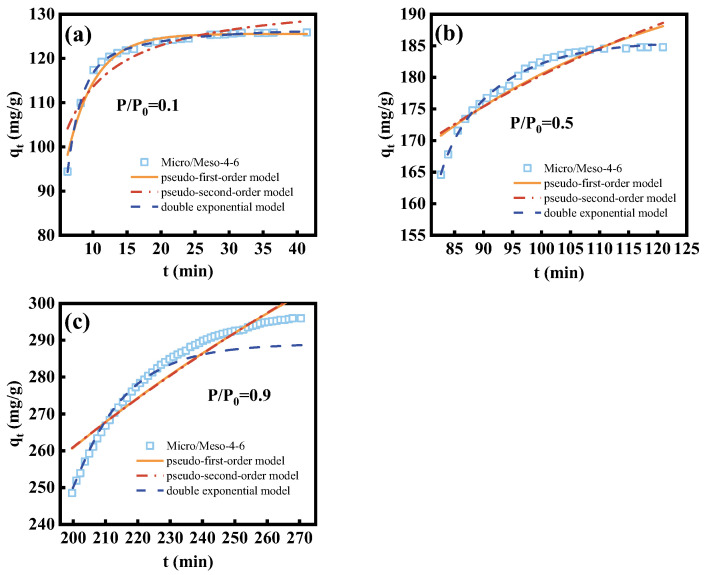
Fitting of pseudo-first-order, pseudo-second-order and double-exponential models for composite adsorbent at different partial pressures.

Adsorption experiments were conducted on the composite adsorbents at different relative pressures (P/P_0_ = 0.1, 0.5, and 0.9) using a multistation gravimetric gas/vapor adsorption analyzer supplied by Beijing Best Instruments Technology Co., Ltd. (Beijing, China). The obtained water adsorption curves were fitted with kinetic models, and the fitting results are shown in Figure 9, with the corresponding parameters listed in Table 4 and Table 5. As shown in Table 4, the R^2^ values of the pseudo-first-order and pseudo-second-order models for Micro/Meso-4-6 are lower than those of the double-exponential model at all relative pressures, indicating that the double-exponential model better describes the adsorption kinetics over the full humidity range. This suggests that the adsorption process is governed by multiple kinetic steps rather than a single rate-controlling mechanism. The double-exponential model reveals a typical two-stage adsorption process consisting of a fast adsorption stage and a slow adsorption stage. The parameters k_3_ and k_4_ correspond to the apparent rate constants of the fast and slow adsorption stages, respectively [39]. As shown in Table 5, the fast-stage rate constant k_3_ remains stable at approximately 0.5, indicating a rapid initial kinetic response of the composite adsorbent. Combined with the hierarchical pore structure of the composite adsorbent, this stage can be mainly attributed to rapid surface adsorption and mesopore diffusion. In contrast, the slow-stage rate constant k_4_ varies more noticeably with material type and relative pressure, suggesting that the later adsorption stage is more strongly affected by pore structure and adsorption conditions. This stage is mainly associated with micropore filling and diffusion limitations within narrow pore channels. As adsorption proceeds, water molecules gradually penetrate into the microporous regions, where stronger adsorption potentials and restricted diffusion pathways lead to slower mass transfer and longer equilibration times. Overall, the double-exponential fitting results reveal a two-stage adsorption process involving fast and slow adsorption steps. The physical interpretation of the two adsorption stages is also consistent with the diffusion coefficient analysis shown in Table 6. This indicates that different pore structures in the composite adsorbents may play distinct roles during adsorption, while the synergy between strongly hydrophilic adsorption sites and mesoporous transport channels contributes to improved overall adsorption performance. It should be noted that the kinetic parameters in this study were obtained using the linearized forms of the PFO and PSO models, which are commonly adopted for comparative adsorption analysis in related studies. Although nonlinear regression may provide a more statistically rigorous parameter estimation and reduce potential bias associated with linearization, the present analysis mainly focuses on comparative kinetic behavior and adsorption-mechanism interpretation. Further studies incorporating nonlinear fitting and comprehensive statistical evaluation will be considered in future work.

The diffusion coefficients of microporous and mesoporous materials under different relative pressures are summarized in Table 6, which clearly reveals the decisive influence of pore structure on mass transfer behavior and its variation law with humidity. In the low relative pressure region (P/P_0_ = 0.1), the order of diffusion coefficients is microporous material (5.15 × 10^−5^) > mesoporous material (2.67 × 10^−5^). At this time, gas-phase diffusion dominates in the micropores, and the strong adsorption potential accelerates the migration of water molecules, resulting in the fastest mass transfer. In the medium relative pressure region (P/P_0_ = 0.5), the diffusion coefficient of mesoporous material (1.96 × 10^−5^) is much higher than that of microporous material (1.08 × 10^−7^). At this stage, the effective space in the micropores is sharply reduced due to adsorption accumulation, and the diffusion coefficient decreases by 2 orders of magnitude compared with the low-pressure region. In contrast, the mesoporous materials still rely on surface diffusion, and the diffusion coefficient remains at the order of 10^−5^, making the mass transfer rate exceed that of microporous materials. In the high relative pressure region (P/P_0_ = 0.9), the mesoporous materials form liquid-phase channels due to capillary condensation, and the diffusion coefficient reaches its peak; although the diffusion coefficient of microporous materials slightly rebounds, it is still 2 orders of magnitude lower than that of mesoporous materials. In summary, microporous materials are dominant in mass transfer under low relative pressure, while mesoporous materials are dominant under high relative pressure.

### 3.9. Adsorption Isotherms and Model Fitting

**Figure 10:**
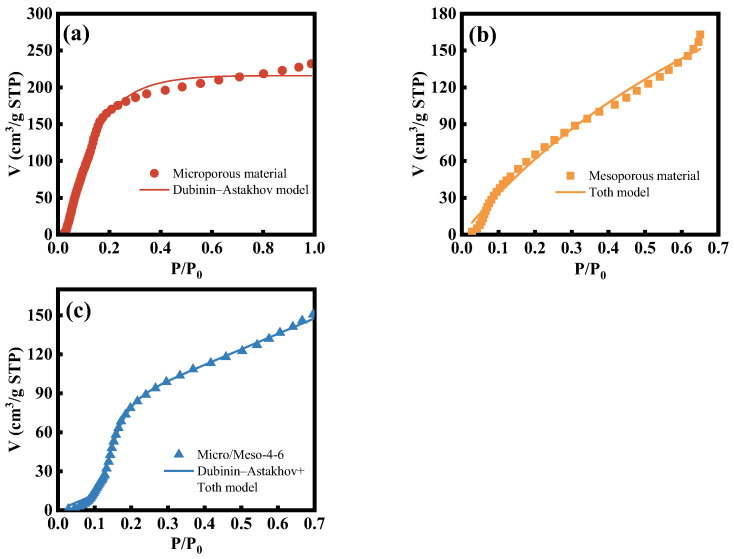
Adsorption isotherms and model fitting of (**a**) Microporous material, (**b**) Mesoporous material, and (**c**) Microporous/mesoporous composite adsorbent.

To elucidate the water vapor adsorption mechanism of microporous/mesoporous composite adsorbents, model fitting and mechanistic analysis were separately conducted on the adsorption isotherms of pure microporous materials, pure mesoporous materials, and the composite adsorbent. The fitting results are displayed in Figure 10, and the corresponding fitting parameters are summarized in Table 7 and Table 8. The water vapor adsorption isotherm of the pure microporous material can be satisfactorily fitted by the Dubinin–Astakhov (D–A) model, with a correlation coefficient R^2^ higher than 0.99. This indicates that its adsorption process is dominated by micropore volume filling, featuring a highly ordered pore structure and uniform adsorption potential distribution.

By contrast, the pure mesoporous material conforms better to the Toth model, and its adsorption behavior is governed mainly by heterogeneous surface adsorption, which is strongly influenced by the energy distribution of surface adsorption sites. The pore size of the mesoporous material is predominantly in the range of 2–50 nm, which is far larger than the kinetic diameter of water molecules, thus giving rise to no obvious micropore volume-filling effect. The adsorption process mainly depends on the interactions between water molecules and surface adsorption sites such as hydroxyl groups, and the energy distribution of these surface sites presents remarkable heterogeneity (e.g., the coexistence of strongly adsorptive hydroxyl sites and weakly adsorptive ordinary sites). For the microporous/mesoporous composite adsorbent system, a single adsorption model can hardly accurately describe the dual adsorption mechanisms simultaneously—micropore volume filling in the microporous phase and heterogeneous surface adsorption in the mesoporous phase. Therefore, an additive model constructed by combining the D–A model and the Toth model was adopted to fit and analyze the adsorption isotherms. This additive fitting approach not only delivers excellent fitting performance (coefficient of determination R^2^ > 0.99) but also allows the quantitative deconvolution of the contributions from microporous and mesoporous components to the total adsorption capacity. Specifically, the characteristic parameter of the D–A model (V_0_) corresponds to the adsorption contribution of the microporous component, while the Toth model parameter (V_m_) characterizes that of the mesoporous component. On the basis of the fitting results, the synergistic mechanism of the hierarchical pore structure in the dehydration process can be clearly revealed: the microporous component enables highly efficient selective adsorption and rapid capture of trace water vapor, whereas the mesoporous component significantly boosts the overall adsorption capacity of the system. Their synergistic effect endows the composite adsorbent with outstanding adsorption selectivity and high uptake capacity, rendering it more compatible with the practical demands of industrial deep dehydration.

### 3.10. Adsorption Performance

**Figure 11:**
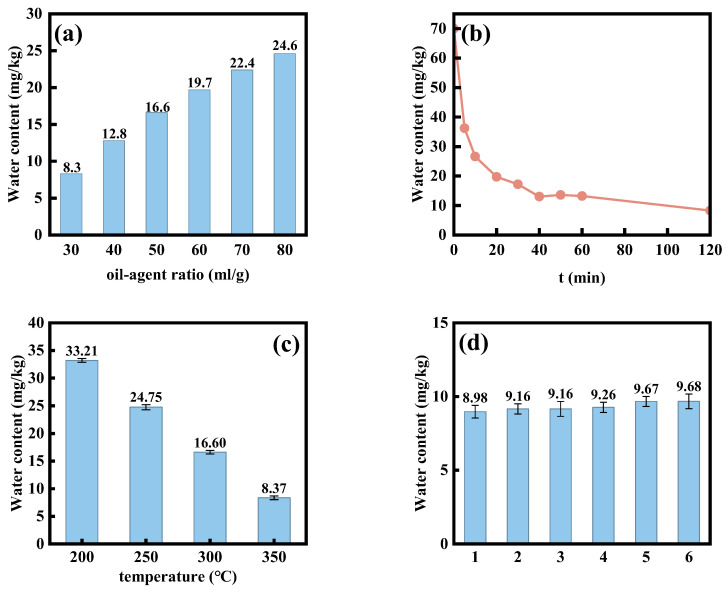
Effects of (**a**) solvent-to-adsorbent ratio, (**b**) contact time, (**c**) regeneration temperature and (**d**) regeneration cycles on the dehydration performance of Micro/Meso-4-6.

To investigate the effects of contact time and solvent-to-adsorbent ratio on dehydration performance, the Micro/Meso-4-6 with the best dehydration effect was selected for batch dehydration experiments. When investigating the effect of contact time, the solvent-to-adsorbent ratio was set to 30 mL/g; when investigating the effect of solvent-to-adsorbent ratio, the adsorption time was set to 18 h. As shown in Figure 11a, the final water content of THF increases with the increase in the oil-agent ratio. This is because the number of active sites of the adsorbent is fixed, and the increase in the s solvent-to-adsorbent ratio leads to an increase in the total amount of water molecules that need to be treated per unit mass of adsorbent; at the same time, the increase in solvent volume reduces the contact probability between water molecules and the adsorbent, thereby weakening the adsorption efficiency. When the solvent-to-adsorbent ratio is 30 mL/g, the final water content can be maintained below 10 mg/kg. Therefore, it was determined as the optimal oil-agent ratio. As shown in Figure 11b, the dehydration rate is fast in the initial stage of adsorption, and the water content of THF has decreased to 36 mg/kg after 5 min of contact; subsequently, the adsorption rate gradually decreases, and further decreases to about 8 mg/kg after 120 min. This indicates that the composite adsorbent can achieve rapid water reduction in a short time and finally reach the deep dehydration level.

To investigate the regeneration performance of the adsorbent, the deactivated adsorbent was heated at different temperatures for 4 h for regeneration, with the solvent-to-adsorbent ratio fixed at 30 mL/g and the adsorption time fixed at 18 h. As shown in Figure 11c, the final water content of THF decreases with the increase in regeneration temperature. After regeneration at 200 °C, 250 °C, and 300 °C, the water content is still higher than 10 mg/kg, indicating that these temperatures are not sufficient to fully restore the dehydration performance. When the temperature is increased to 350 °C, the water content decreases significantly to 8.3 mg/kg, which is equivalent to that of the fresh adsorbent. In the regeneration experiment, to ensure that the adsorbed water in the adsorbent is completely removed, 350 °C was selected as the appropriate regeneration temperature. As shown in Figure 11d, after six adsorption-regeneration cycles, the composite adsorbent still maintains stable dehydration performance, and the final water content of THF consistently remained below the industrial requirement. In addition, no obvious changes in the appearance or aggregation behavior of the adsorbent particles were observed after repeated regeneration. These results indicate that the composite adsorbent possesses good regeneration ability and operational stability under the applied regeneration conditions.

### 3.11. Proposed Adsorption Mechanism

Based on the structural characterization, liquid-phase dehydration tests, and water-vapor adsorption behavior, the superior performance of the composite adsorbents can be attributed to the cooperative effects of the microporous and mesoporous domains. The LTA-type microporous phase provides size-selective adsorption sites for water and enables low final water contents, whereas the mesoporous phase supplies lower-resistance transport pathways that improve accessibility and partially alleviate diffusion limitations in liquid THF. The hierarchical structure therefore helps balance selectivity and mass transfer, leading to better dehydration performance than either single-component material alone. The detailed evolution of THF-water associates inside the mesopores remains to be clarified by in situ spectroscopy or molecular simulation.

## 4. Conclusions

A hierarchical microporous/mesoporous composite adsorbent was prepared by extrusion molding. Structural characterization results show that the composite adsorbent retains both the microporous structural characteristics of LTA-type zeolites and the pore channel characteristics of mesoporous materials. Static dehydration results demonstrate that at a microporous/mesoporous mass ratio of 4:6, the composite shows optimal dehydration performance, which can reduce the water content of tetrahydrofuran from 70 mg/kg to 8.3 mg/kg, and exhibits better deep dehydration ability than commercial 3A zeolite. Water vapor adsorption, kinetic and isotherm fitting, competitive adsorption, and regeneration experiments indicate that the hierarchical structure improves water accessibility and that the adsorption kinetics are well described by the double-exponential model. After regeneration at 350 °C, the adsorbent substantially recovered its original performance and maintained stable dehydration behavior over six cycles. These results show that hierarchical microporous/mesoporous design is an effective strategy for deep dehydration of THF.

## Figures and Tables

**Table 1:** Specific surface area and pore volume data of microporous material based on CO_2_ adsorption–desorption measurement.

Adsorbent	S_CO2_ (m^2^/g)	V_HK_ (cm^3^/g)	D (nm)
Microporous material	390	0.11	0.35

**Table 2:** Specific surface area and pore volume data of the adsorbents based on N_2_ adsorption–desorption measurements.

Absorbent	S_BET_ ^a^	S_Langmuir_ ^b^	V_Total_ ^c^	V_Micro_ ^d^	V_Meso_
Microporous material	53	45	0.05	0.01	0.04
Micro/Meso-8-2	78	66	0.17	0.03	0.14
Micro/Meso-6-4	116	102	0.21	0.04	0.17
Micro/Meso-4-6	168	145	0.26	0.06	0.20
Micro/Meso-2-8	230	207	0.35	0.08	0.27
Mesoporous material	281	256	0.39	0.10	0.29

^a^ BET specific surface area. ^b^ Surface area was determined utilizing the BET equation. ^c^ Micropore volume calculated by HK/SF. ^d^ Total pore volume was computed via the BJH method. It should be noted that the micropore-volume values derived from N_2_ adsorption correspond only to the N_2_-accessible microporous fraction estimated by the t-plot method. Because the kinetic diameter of N_2_ is close to the pore opening of K-LTA, the true ultramicroporosity of the LTA component is better reflected by the CO_2_ adsorption results shown in Table 1.

**Table 3:** Multi-component adsorption breakthrough result.

Component	5% Breakthrough Point (s·g^−1^)	50% Half-Saturation Point (s·g^−1^)	95% Saturation Point (s·g^−1^)	Adsorption Uptake (mL·g^−1^ STP)	THF/H_2_O Separation Factor
THF	2159.7	2664.7	3019.5	7.433	0.1652
H_2_O	7850.3	8655.4	11,384.7	234.008	

**Table 4:** R^2^ data obtained by fitting the three models.

Adsorbent	P/P_0_	R^2^ (Pseudo-First-Order Model)	R^2^ (Pseudo-Second-Order Model)	R^2^ (Double-Exponential Model)
Micro/Meso-4-6	0.1	0.9687	0.8325	0.9987
	0.5	0.8516	0.8210	0.9937
	0.9	0.9030	0.8970	0.9999

**Table 5:** Fitting parameters of the double-exponential model.

Adsorbent	P/P_0_	Experimental Equilibrium Adsorption Values (mg/g)	Fitted Equilibrium Adsorption Values (mg/g)	k_3_ (1/s)	k_4_ (1/s)
Micro/Meso-4-6	0.1	125.9	126.6	0.5	0.083
	0.5	184.8	185.7	0.5	0.096
	0.9	296.0	298.7	0.5	0.042

**Table 6:** Diffusion coefficients of different adsorbents.

Diffusion Coefficients (cm^2^/s)	P/P_0_		
	0.1	0.5	0.9
Microporous material	5.15 × 10^−5^	1.08 × 10^−7^	2.86 × 10^−7^
Mesoporous material	2.67 × 10^−5^	1.96 × 10^−5^	4.85 × 10^−5^

**Table 7:** The R^2^ obtained from fitting.

Adsorbent	R^2^
Microporous material	0.9946
Mesoporous material	0.9895
Micro/Meso-4-6	0.9982

**Table 8:** The fitting parameters of the D–A + Toth model.

Adsorbent	V_0_ (cm^3^/g STP)	V_m_ (cm^3^/g STP)
Micro/Meso-4-6	64.23	333.61

## Data Availability

The original contributions presented in this study are included in the article. Further inquiries can be directed to the corresponding author.
